# Glycogen synthase kinase-3β inhibition induces nuclear factor-κB-mediated apoptosis in pediatric acute lymphocyte leukemia cells

**DOI:** 10.1186/1756-9966-29-154

**Published:** 2010-11-26

**Authors:** Yanni Hu, Xiaoyan Gu, Ruiyan Li, Qing Luo, Youhua Xu

**Affiliations:** 1 Laboratory of Oncology, Affiliated Children's Hospital, Chongqing Medical University, No.136, Zhongshan 2nd Road, Yuzhong District, Chongqing 86 400014, China; 2 Department of Hematology, Affiliated Children's Hospital, Chongqing Medical University, No.136, Zhongshan 2nd Road, Yuzhong District, Chongqing 86 400014, China

## Abstract

**Background:**

Molecular therapies that target genetic abnormalities in leukemic cells and their affected signaling pathways have been emerging in pediatric acute lymphoblastic leukemia (ALL). Glycogen synthase kinase-3β (GSK-3β) has recently been found to positively regulate the activity of nuclear factor-κB (NF-κB). Here, we investigated the relationship between GSK-3β inhibition and NF-κB in apoptosis of pediatric primary leukemia cells obtained from 39 newly diagnosed ALL children in China.

**Methods:**

Bone marrow mononuclear cells (BMMC) were isolated by density gradient centrifugation from the heparinized aspirates of children with ALL. We used immunofluorescence staining to detect nuclear GSK-3β in these cells. After treatment with chemically distinct GSK-3β inhibitors in vitro, NF-κB transcriptional activity was identified by means of western blotting and electrophoretic mobility shift assay (EMSA). NF-κB-mediated apoptosis was detected by Annexin V-PE/7-AAD double-staining flow cytometry. The expression level of the *survivin *gene was detected by reverse-transcriptase polymerase chain reaction (RT-PCR).

**Results:**

GSK-3β significantly accumulates in the nuclei of ALL cells than in the nuclei of control cells. Cell death induced by GSK-3β inhibition in ALL cells was mediated by a downregulation of NF-κB p65 transcriptional activity. GSK-3β inhibition significantly decreased the expression of the NF-κB target gene *survivin*.

**Conclusions:**

These results indicate that inhibition of GSK-3β downregulates the NF-κB activation pathway, leading to suppression of the expression of an NF-κB-regulated gene and promotion of apoptosis in ALL cells in vitro. Furthermore, our findings suggest that GSK-3β or NF-κB is a potential therapeutic target in the treatment of pediatric ALL.

## Introduction

Acute lymphocytic leukemia (ALL) is the most common malignancy diagnosed in children, and it accounts for approximately one-third of all pediatric cancers. Although contemporary treatments cure more than 80% of children with ALL, some patients require intensive treatment and many patients still develop serious acute and late complications because of the side effects of the treatments [1]. Therefore, new treatment strategies are needed to improve not only the cure rate but also the quality of life of these children [2].

Glycogen synthase kinase-3 (GSK-3) is a serine/threonine protein kinase, whose activity is inhibited by a variety of extracellular stimuli including insulin, growth factors, cell specification factors, and cell adhesion [3-5]. Two homologous mammalian GSK-3 isoforms are encoded by different genes, *GSK-3α *and *GSK-3β*. Recently, GSK-3 has been recognized as a key component of a diverse range of cellular functions essential for survival [6]. Fibroblasts from GSK-3β-deficient embryos were sensitized to apoptosis and showed reduced nuclear factor-κB (NF-κB) function [7]. Furthermore, it has been shown that GSK-3β is a prosurvival factor in pancreatic tumor cells, partly through its ability to regulate the NF-κB pathway [8]. These findings suggest a role for GSK-3β (but not GSK-3α) in the regulation of NF-κB activation. Recent experimental evidence has suggested that inhibition of GSK-3β abrogates NF-κB binding to its target gene promoters through an epigenetic mechanism and enhances apoptosis in chronic lymphocytic leukemia (CLL) B cells ex vivo [9]. Consequently, inhibition of GSK-3β activity has been proposed to play a role in the regulation of the NF-κB signaling pathway that elicits cellular survival responses.

However, little is currently known about the significance of GSK-3β to pediatric ALL cell survival. ALL initiates and progresses in the bone marrow (BM). In the present study, we demonstrated that GSK-3β accumulates in the nuclei of primitive pediatric ALL cells from the BM. GSK-3β inhibition leads to suppression of NF-κB transcriptional activity and induces apoptosis through the transcriptional downregulation of the *survivin *gene.

## Methods

### Primary cells

Fresh ALL samples were obtained from 39 children with newly diagnosed acute lymphoblastic leukemia, with 11 normal BM samples as control, in Affiliated Children's Hospital, Chongqing Medical University. The diagnosis of ALL was based on morphology, immunology, cytogenetic, and molecular classification. The informed consent was obtained from parents, guardians, or patients (as appropriate).

### Isolation of leukemia cells and cell culture

Bone marrow mononuclear cells (BMMC) were isolated from heparinized aspirates by Ficoll-Hypaque density gradient centrifugation within 24 h after sampling. To remove adherent cells, BMMC were suspended in RPMI 1640 medium supplemented with 20% fetal calf serum (FCS) and incubated in plastic dishes at 37°C for 24 h before collection of nonadherent cells. These ALL cells were then either used immediately for the laboratory studies described below or cryopreserved in RPMI 1640 medium with 20% FCS and 10% dimethyl sulfoxide (DMSO) and stored in liquid nitrogen until use. If necessary, leukemic samples were further enriched to more than 90% leukemic blasts by removing nonmalignant cells with immunomagnetic beads [10].

### Reagents and antibodies

The GSK-3β inhibitors SB216763, and lithium chloride (LiCl) were obtained from Sigma, USA. A 20 mg/ml solution of SB216763 was prepared in dimethyl sulfoxide (DMSO), stored in small aliquots at -20°C, and then thawed and diluted in cell-culture medium as required. LiCl was dissolved in RPMI 1640 and used at final concentrations of 5 and 10 mM. The high-quality fetal bovine serum and RPMI 1640 medium were products of Gibco Company, USA. RNAiso Plus, Reverse Transcription PCR kits, and primers were products of TaKaRa Biotechnology, Dalian, China. DyLight 549-conjugated goat anti-rabbit IgG and Hoechst 33342 were obtained from CWBio, Beijing, China. Antibodies for immunoblot analysis were obtained from the following suppliers: GSK-3β and NF-κB p65 from Cell Signaling Technology, USA; survivin, β-actin, histone, and goat anti-rabbit IgG-horseradish peroxidase (HRP) from Santa Cruz Biotechnology, CA.

### Analysis of GSK-3β expression in ALL cells by immunofluorescence microscopy

BMMC that had been attached to glass slides by cytocentrifugation (StatSpin InC, USA) were fixed with 4% paraformaldehyde in phosphate-buffered saline (PBS), permeabilized with 0.3% Triton X-100 for 10 min at room temperature, and blocked with 3% bovine serum albumin (BSA) for 30 min. The cells were subsequently subjected to immunofluorescence staining to detect GSK-3β expression with the same primary antibody (diluted 1:200) used for immunoblotting. After a rinse in PBS, cells were incubated with secondary DyLight 549-conjugated goat anti-rabbit IgG antibody. Nuclei were counterstained with Hoechst 33342. SlowFade mounting medium was used. Images were acquired using the Leica Application Suite on a fluorescence microscope (Olympus, Japan) equipped with a 40 ×/0.75 oil DIC objective.

### Western blotting

Leukemic cells (1 × 10^7^) undergoing different treatments were rinsed with PBS and lysed in buffer. Nuclear/Cytosolic fractionation was performed using nuclear-cytosol extraction kit (KENGEN Biotechnology, Nanjing, China) according to the manufacturer's instructions. Protein sample concentration was quantified by the BCA method and an equal amount (30 μg of cytosolic or nuclear protein extract) of proteins was loaded in each well of a 10% SDS polyacrylamide gel. Cell extracts were separated by polyacrylamide gel electrophoresis (PAGE), and transferred to polyvinylidene difluoride membrane (PVDF). Primary antibodies against GSK-3β, NF-κB p65, survivin, β-actin, and histone were used. HRP-conjugated anti-IgG was used as the secondary antibody. Western blot band intensities were quantified using Quantity One software (Bio-Rad Laboratories, Inc., USA).

### Electrophoretic mobility shift assays (EMSA) for NF-κB

Nuclear lysates were prepared and protein concentrations were measured by the BCA protein assay according to the manufacturer's manual. Equivalent amounts of nuclear extract proteins (2 μg) were preincubated in 1 μl of binding buffer for 20 min at room temperature. Then, a biotin-labeled oligonucleotide probe was added, and the reaction mixture was incubated for 20 min at room temperature. For reactions involving competitor oligonucleotides, the unlabeled competitor and the labeled probes were premixed before addition to the reaction mixture. The samples were analyzed on 6.5% acrylamide gels and electrophoresis was carried out at 180 V for 70 min. Gel contents were transferred to binding-membrane, dried, incubated with streptavidin-HRP, and exposed with an intensifying screen.

### Reverse-transcriptase polymerase chain reaction analysis (RT-PCR)

Total RNAs were extracted according to the manufacturer's instructions and were reverse-transcribed using the PrimeScript RT reagent Kit (TaKaRa, Dalian, China). Of a 20 μl cDNA reaction, 5 μl was used as template for amplification with the following specific primers. For human survivin forward: 5'-TCCACTGCCCCACTGAGAAC-3' and reverse 5'-TGGCTCCCAGCCTTCCA-3'; for human GAPDH forward: 5'-CAGCGACACCCACTCCTC-3' and reverse 5'-TGAGGTCCACCACCCTGT-3'. The PCR was performed with the first denaturation step at 94°C for 5 min, and 35 cycles of denaturation at 94°C for 1 min, annealing at 60°C for 30 s, and extension at 72°C for 1 min. The PCR reaction products were detected with gel electrophoresis and ultraviolet transillumination.

### Apoptosis assay

BMMC (1 × 10^6 ^cells) were exposed to GSK-3β inhibitors or a matched concentration of diluents. After the treatment, cells were rinsed twice in cold PBS, resuspended in binding buffer, and then analyzed for apoptosis level by a PE-labeled Annexin-V/7-AAD assay. These cells were directly analyzed in a FACScan (BD FACS Calibur Co., USA) with a sample size of at least 10,000 cells gated on the basis of forward and side scatter. Storing and processing of data were accomplished using FACScan software.

### Statistical analysis

Results are expressed as mean ± standard deviation. Statistical analysis was conducted using SPSS 15.0 software. Differences between groups were examined for statistical significance using a one-way analysis of variance and Student's *t*-test; P values less than 0.05 were considered statistically significant.

## Results

### GSK-3β accumulated in the nucleus of primary ALL cells

**Figure 1:**
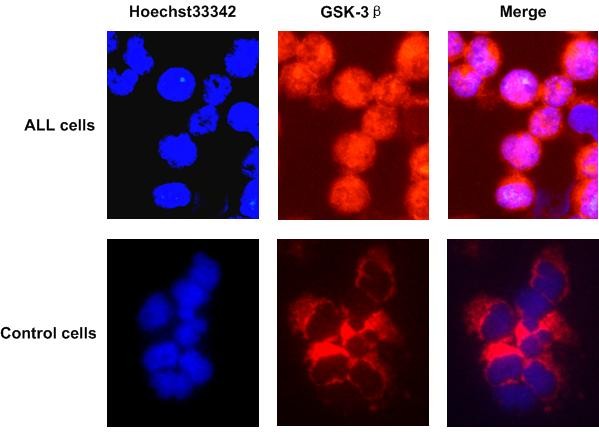
**Immunofluorescence staining of GSK-3β in ALL cells**. Bone marrow samples were obtained from children with ALL and from control patients. GSK-3β was probed with Dylight 549-labeled anti-rabbit secondary antibody (red fluorescence) and nuclei were counterstained with Hoechst 33342 (blue fluorescence). Nuclear accumulation of GSK-3β in ALL cells was detected, whereas only cytoplasmic expression of GSK-3β was observed in control cells.

Using immunofluorescence staining, we identified the localization of GSK-3β in ALL BMMC in 8 children with ALL. As shown in Figure 1, we found nuclear accumulation of GSK-3β in 6 primary pediatric ALL BMMC samples, whereas it was not detected in the nucleus of control BMMC.

### Inhibition of GSK-3β suppressed the binding of NF-κB to the DNA in ALL cells

**Figure 2:**
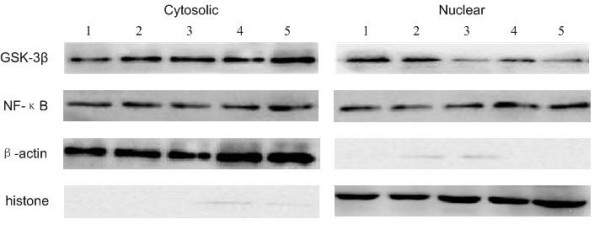
**Detection of GSK-3β and NF-κB protein expression, after 48 h of treatment with GSK-3β inhibitors, by western blot assay**. ALL cells were cultured in the presence of LiCl (10 mM) or SB216763 (10 μM) for 48 h. Cytosolic and nuclear fractions were prepared from the indicated samples. β-Actin and histone were used as markers for the purity of the cytosolic and nuclear fractions, respectively. GSK-3β inhibition led to depletion of GSK-3β nuclear pool in ALL cells, whereas nuclear levels of NF-κB p65 remained unchanged. The data shown are representative of 3 independent experiments. 1: untreated ALL cells; 2: ALL cells treated with NaCl; 3: ALL cells treated with LiCl (10 mM); 4: ALL cells treated with DMSO; 5: ALL cells treated with SB216763(10 μM).

**Figure 3:**
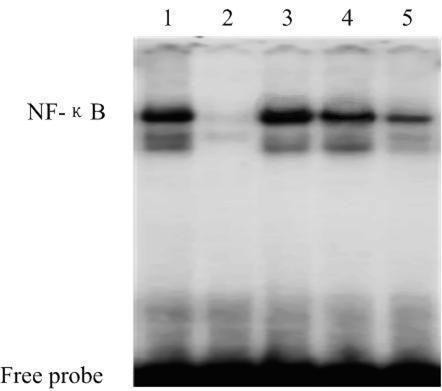
**Effects of GSK-3β inhibitors on DNA binding activity of NF-κB in nuclear extracts of ALL cells**. After 48 h of treatment with GSK-3β inhibitors, ALL cells nuclear extracts were prepared and assayed for NF-κB activation by EMSA as described under "Methods." GSK-3β inhibitors resulted in a reduction in NF-κB DNA binding activity when compared to control condition (untreated ALL cells). The data shown are representative of 3 independent experiments. 1: negative control; 2: positive control; 3: untreated ALL cells; 4: ALL cells treated with LiCl (10 mM); 5: ALL cells treated with SB216763 (10 μM).

GSK-3β has been shown to play a critical role in NF-κB-mediated survival of cancer cells. The aberrant accumulation of GSK-3β in nuclei of ALL cells prompted us to examine the effect of GSK-3β inhibition on NF-κB activity. Using primary ALL cells, we tested ex vivo the effect of 2 chemically distinct small-molecule inhibitors of GSK-3β: SB216763 (ATP-competitive, arylindolemaleimide) [11], and LiCl (non-ATP-competitive) [12]. Forty-eight hours after GSK-3β inhibitors treatment, we estimated the level of GSK-3β inhibition by detection of the cytosolic/nuclear level of GSK-3β by western blot. We found that both the distinct GSK-3β inhibitors can decrease the level of GSK-3β in nuclear extracts of ALL cells (Figure 2). With the same treatments, nuclear levels of NF-κB p65 in ALL cells were not significantly changed (Figure 2). To further investigate the role of GSK-3β in the regulation of NF-κB activity, we detected NF-κB DNA binding activity by EMSA. The data show that GSK-3β inhibition in ALL cells decreased the binding of NF-κB p65 to its target gene promoter (Figure 3). Taken together, these results suggest that GSK-3β affects NF-κB activity at the transcriptional level in pediatric ALL cells.

### Pharmacologic inhibition of GSK-3β induced apoptosis in ALL cells

**Figure 4:**
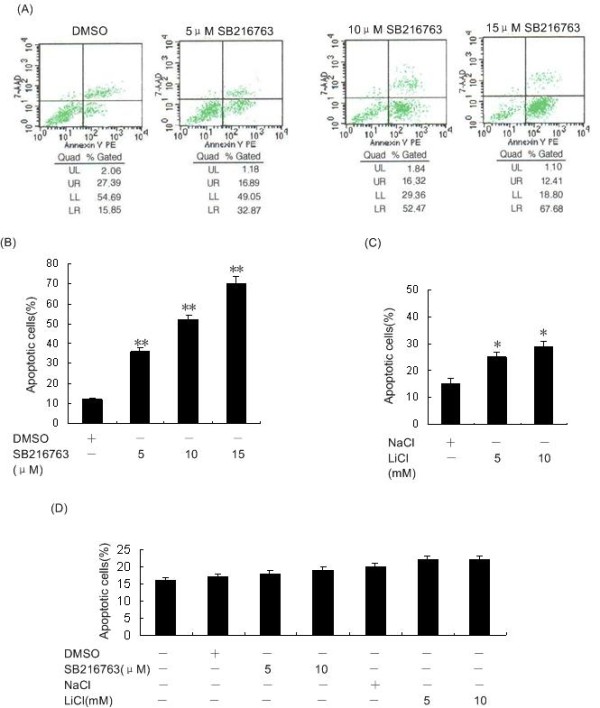
**Inhibition of GSK-3β induces apoptosis in ALL but not control cells**. (A) ALL cells were treated for 48 h with DMSO or SB216763 at indicated concentrations. Cells were assayed for apoptosis using Annexin V-PE/7-AAD staining by flow cytometry. (B) We found that inhibition of GSK-3β in ALL cells consistently resulted in a dose-dependent increase in the number of apoptotic cells. (C) ALL cells were treated for 48 h with NaCl or LiCl at indicated concentrations, then assayed for apoptosis using Annexin-V-PE/7-AAD staining as determined by flow cytometry. (D) Each GSK-3β inhibitor was added to control BMMC at the initiation of the culture at a variety of concentrations as indicated on the graph. LiCl and SB216763 had no significant effect on cell apoptosis in normal BMMC. Columns, mean; bars, SD. *P < 0.05, **P < 0.01 vs. control. All assays were performed in triplicate.

Since NF-κB is a potential target of GSK3β-dependent cell survival pathway, we detected apoptotic cells as an Annexin-V^+^/7-AAD^+ ^population within DMSO or SB216763-treated malignant cells cultured ex vivo from each of the 11 patients with ALL by using Annexin-V staining and flow cytometry. Although the mean number of apoptotic cells was 12% in DMSO-treated ALL cells, the apoptotic cell fraction in the SB216763-treated cells was significantly higher; the mean number of apoptotic cells reached 36% (SB216763, 5 μM), 52% (SB216763, 10 μM) and 70% (SB216763, 15 μM) after 48 h of exposure (Figure 4A, B; P < 0.001). It demonstrated that the number of apoptotic cells dose-dependently increased with SB216763 treatment. We also evaluated the apoptotic effect of LiCl, another GSK-3β inhibitor, on ALL cells. LiCl, at subtoxic concentrations, induced NF-κB-mediated apoptosis in a dose-dependent manner (Figure 4C; P < 0.05). These results confirmed that GSK-3β suppression leads to ALL apoptosis.

### GSK-3β inhibitors had no significant effect on cell apoptosis in normal BMMC

To further evaluate whether GSK-3β inhibition specifically induced apoptosis in ALL cells, we examined the effect of GSK-3β inhibitors on normal BMMC. GSK-3β inhibition was previously shown to preserve umbilical cord blood stem cell activity [13]. However, consistent with the localization of GSK-3β in the nuclei of normal BMMC, we found that the number of apoptotic cells in normal BMMC was not significantly changed in the presence or absence of GSK-3β inhibitors after 48 h of treatment (Figure 4; P > 0.05). The results obtained with GSK-3β inhibition in normal progenitors versus ALL cells provide evidence of a significant therapeutic selectivity.

### Pharmacologic inhibition of GSK-3β decreased NF-κB-mediated expression of an antiapoptotic molecule in ALL cells

**Figure 5:**
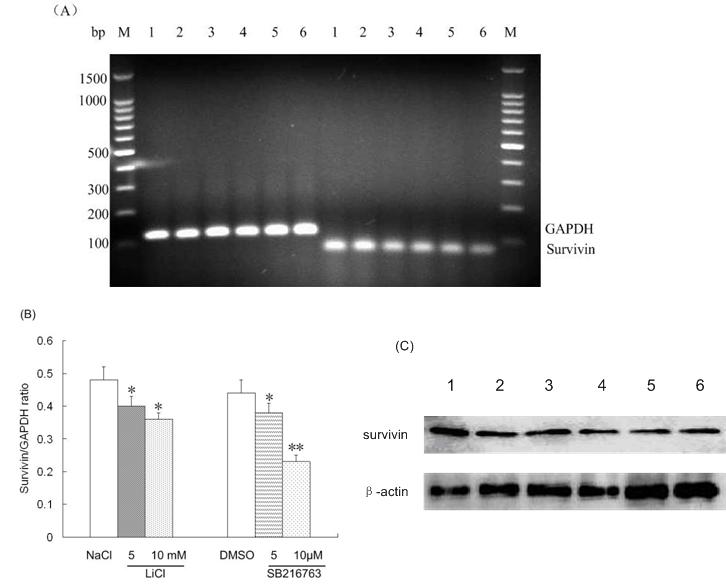
**Inhibition of GSK-3β decreased NF-κB-mediated expression of the antiapoptotic molecule survivin in ALL cells**. Cells from patients with ALL were treated with controls (NaCl/DMSO) or GSK-3β inhibitors (LiCl/SB216763) for 48 h. (A) The cell pellet was collected and RNA was obtained, then RT-PCR analysis was performed. (B) *Survivin *mRNA levels were normalized to GAPDH levels in each group. NaCl (48 ± 4)% vs. LiCl (5 mM (40 ± 5)%, 10 mM (36 ± 3)%); DMSO (44 ± 5)% vs. SB216763 (5 μM (38 ± 4)%, 10 μM (23 ± 3)%). (C) Total cell lysates were separated by SDS-PAGE, transferred to PVDF membrane, and immunoblotted with the indicated antibodies. *P < 0.05 vs. controls, **P < 0.01 vs. controls. DNA marker; 1: NaCl; 2: DMSO; 3: LiCl, 5 mM; 4: LiCl, 10 mM; 5: SB216763, 5 μM; 6: SB216763,10 μM.

Pharmacologic inhibition of GSK-3β induced apoptosis in ALL cells, so we further investigated whether inhibition of GSK-3β affects NF-κB-mediated expression of the antiapoptotic gene *survivin *in cells from 10 patients with ALL. We found that inhibition of GSK-3β resulted in decreased mRNA and protein expressions of NF-κB target gene *survivin *in ALL cells relative to control cells (Figure 5). After completion of these experiments, we summarized the data and represented it as a mean value (Figure 5 legend). SB216763 (10 μM) and LiCl (10 mM) treatment resulted in a 47.7% and 25% reduction in *survivin *mRNA levels, respectively. Moreover, the levels of *survivin *mRNA decreased dose-dependently after treatment with both LiCl and SB216763. These results indicate that the inhibition of GSK-3β does not affect the nuclear accumulation of NF-κB p65 but might alter the ability of NF-κB to regulate target gene promoters in ALL cells.

## Discussion

GSK-3β has recently been shown to be a crucial enzymatic regulator of cancer cell survival in human tumorigenesis [14,15]. GSK-3β protein is known to be overexpressed in human prostate [16], pancreatic [17], and colon [14] carcinomas. Normally, GSK-3β is expressed in the cytoplasm of cells. Recent studies have shown that GSK-3β could shuttle from the cytoplasm to the nucleus in pancreatic cancer cell lines and in most poorly differentiated human pancreatic adenocarcinomas [17], and in human CLL B cells [9]. In this study, we found aberrant nuclear accumulation of GSK-3β in cells obtained from children with ALL, whereas GSK-3β was not detected in the nucleus of control cells.

GSK-3β transposition was thought to participate in the regulation of gene transcription through the phosphorylation of transcription factors [18]. NF-κB, an important transcription factor also involved in the regulation of cell proliferation, differentiation, and apoptosis, is deregulated in many human tumors [19,20]. Previous studies have suggested that NF-κB transcriptional activity is regulated by GSK-3β [7]. Genetic depletion of GSK-3β by RNA interference suppresses basal NF-κB transcriptional activity, leading to decreased pancreatic cancer cell proliferation and survival [8]. Recently, it has been demonstrated that GSK-3β positively regulates NF-κB-mediated drug resistance in acute myeloid leukemia (AML) [21]. In this study, we tested ex vivo the effect of 2 chemically distinct small-molecule inhibitors of GSK-3β at subtoxic concentrations: LiCl, a well-known GSK-3β inhibitor, and SB216763, a widely used maleimide-containing GSK-3β inhibitor. Using the pharmacological inhibitors of GSK-3β, we estimated the level of GSK-3β inhibition by detecting the protein levels of GSK-3β in cytosolic and nuclear extracts through western blot assay. In ALL cells, we found that both inhibitors led to depletion of the nuclear pool of GSK-3β, whereas no change was found in the cytoplasm extract. Moreover, we found that GSK-3β inhibition in ALL cells did not prevent NF-κB relocation from the cytoplasm to the nucleus, but the inhibition affected the transcriptional repression of NF-κB, as shown by EMSA analysis. Similar to previous studies [7], our studies on pediatric ALL cells show that NF-κB can be regulated by GSK-3β at the level of the nuclear transcriptional complex.

The exact mechanism by which GSK-3β affects NF-κB transcriptional activity is still unknown. GSK-3β influences NF-κB-mediated gene transcription in pancreatic cancer cells at a point distal to the IκB kinase complex [7]. Recent data have demonstrated that GSK-3β may contribute to p65/p50 binding to the promoters and transcriptional activation of NF-κB in CLL cells by regulating histone modification [6]. However, the underlying mechanism by which GSK-3β regulates p65 NF-κB binding to target gene promoters has not been defined.

NF-κB is known as an important factor of cancer cell survival in human tumorigenesis [22]. In this report, we found that GSK-3β suppression sensitized ALL cells to NF-κB-mediated apoptosis. Both SB216763 and LiCl have been shown to induce ALL cells apoptosis. In addition, we observed that there is no significant effect on apoptosis induced by GSK-3β inhibitors in control BMMC. A relevant finding was that GSK-3β was not detected in the nucleus of control BMMC but was detected in the nuclei of ALL cells. Taken together, our results provide evidence of GSK-3β as a novel potential therapeutic target in the treatment of ALL.

Survivin, which is known to be regulated by NF-κB [23], plays a major role in the suppression of apoptosis [24]. Our previous experiments have shown that the expression of the antiapoptotic gene *survivin *significantly increased in children with newly diagnosed acute leukemia (data not shown). Using malignant cells obtained from children with ALL, we have analyzed the effect of GSK-3β inhibition on NF-κB-dependent gene expression involved in the survival of ALL cells. We found that both SB216763 and LiCl could inhibit the expression of *survivin*, thereby promoting cell apoptosis.

## Conclusions

Our data demonstrated for the first time the involvement of GSK-3β in pediatric ALL cells, and not in adult leukemia cells, although GSK-3β inhibition played a similar role in inducing apoptosis in leukemia cells via in vitro activation of NF-κB. Thus, inhibition of GSK-3β and of its target NF-κB signaling pathway could represent a new promising approach for pediatric ALL therapy.

## Competing interests

The authors declare that they have no competing interests.

## Authors' contributions

YH collected the clinical data and samples, drafted and revised the article critically for important intellectual content. YX directed the conception and design of the study. QL participated in the design of the study. XG and RL assisted in acquisition, analysis and interpretation of data. All authors have seen and approved the final manuscript.
